# Biofilm on the polymer composites - qualitative and quantitative microbiological analysis

**DOI:** 10.1007/s40201-021-00634-9

**Published:** 2021-03-04

**Authors:** Dobrochna Ginter-Kramarczyk, Izabela Kruszelnicka, Michał Michałkiewicz, Przemysław Muszyński, Stanisław Zajchowski, Jolanta Tomaszewska

**Affiliations:** 1 Faculty of Environmental Engineering and Energy, Department of Water Supply and Bioeconomy, Poznan University of Technology, Berdychowo 4, 60-965 Poznań, Poland; 2 Faculty of Chemical Technology and Engineering, Department of Polymer Technology, University of Technology and Life Sciences in Bydgoszcz, Seminaryjna 3, 82-326 Bydgoszcz, Poland

**Keywords:** Moving bed biofilm technology, Wood-polymer composites, Wastewater treatment technologies, Biofilm

## Abstract

**Background:**

Modern technology, which has been getting more and more recognition in the world for the last several years, is the moving bed biofilm reactor (MBBR) technology. Currently, movable biofilters made of basic polymeric materials, polyethylene and polypropylene.

**Methods:**

An innovative solution in the field, mainly because of the large active surface area for biological membrane can be wood polymer composites (WPC). In the research polypropylene (PP) and polyvinyl chloride (PVC) was used as the matrix. Two types of commercial wood flour also, selected from conifers, were selected for the study: Lignocel C 120 with particle sizes in the range of 70 μm–150 μm and L9 with dimensions of 0.8–1.1 mm and wood chips, which are used on an industrial scale for the production of chipboards, were used as a filler. A quantitative and qualitative analysis of newly formed biofilms was performed.

**Results:**

The study showed a direct effect of the filler and its particle size on the susceptibility to the formation of the biofilm of on the composites surface.

**Conclusions:**

Polypropylene PPH 648 T and 40% wt. of L9 type wood flour was the most susceptible to biofilm formation. Pure polypropylene PPH 648 T was the least susceptible material.

## Introduction

The increasingly consumer lifestyle of people and changes in social habits result in new threats to aquatic ecosystems. In consequence, both well-known and regulated substances as well as compounds of unknown structure and properties may enter the sewage treatment plant. The substrates present in complex wastewater mixtures may interact (synergism, antagonism), which in some cases leads to enhancement of their potential toxic effect. Therefore, it is necessary to develop new, more efficient technologies for wastewater treatment. Wastewater is most often treated using biological methods. Such processes rely on the oxidation, transformation and removal of contaminants present in wastewater using microorganisms. Aside from the removal of organic compounds, biogenic elements are also dissipated during treatment of wastewater. Depending on the conditions under which the biological degradation of contaminants occurs, aerobic, hypoxic (anoxic) and anaerobic processes may be distinguished. Microorganisms involved in these processes can be attached to a carrier, forming the so-called biological membrane or living in a tank in the form of so-called activated sludge flocs. Depending on the technology used, biological processes are carried out in biological beds or bioreactors [1].

Regardless of the type of biological bed used, the mechanism of contaminant removal is identical. A biological membrane which consists of microorganisms is developed on the surface of the bed filling (e.g. plastic moldings). Wastewater is delivered to the bed with a fixed, controlled flow rate and contact between organic compounds as well as biogenic elements present in the wastewater and microorganisms occurs immediately. Living organisms absorb and consume dissolved organic compounds for their life processes, which results in the increase of their mass [1–3].

Some chemical compounds are retained on the surface of the biological membrane layer. As such, the biofilm acts as a semi-selective and semi-permeable biological membrane. Atmospheric oxygen plays an important role because it penetrates the biofilm layer and intensifies the processes of biological degradation carried out by settled microorganisms in the outer part called the aerobic layer. An anaerobic layer is formed in the inner part, in which substances are decomposed in the framework of anaerobic processes. Colonization of various surfaces by microorganisms is possible due to their adhesive properties and the structure of the formed biofilm is stabilized by the secreted extracellular polymeric substances (EPS) [4–6]. The biofilm is formed by complex multicellular structures in which numerous microbial cells surround the mucus layer. Cells of microorganisms which form the biofilm specialize in performing various functions in enzymatic processes and exhibit different characteristics compared to free-living cells. The design of such clusters protects microorganisms against the adverse effects of external factors and increases their resistance to changes in pH and temperature. Therefore, a biofilm can function under conditions in which the survival of individual cells would be difficult and, in many cases, even impossible [7–9]. The formation of a biofilm is a multistep process. On the one hand, it is conditioned by the properties of microorganisms which from it, on the other hand, it also depends on the structure and properties of colonized materials or a colonized host, which can be another living organism. Extracellular polymers produced by microbes which form a biofilm, lipopolysaccharides and proteins of the cell wall, as well as extracellular structures such as fimbriae and cilia play a special role in adhesion process. The structure of the surface, all its defects and roughness also facilitate colonization [10, 11]. The mechanism of biofilm formation is not yet fully understood. During the process of its formation, various phases can be noticed – including reversible adhesion of microorganisms, irreversible adhesion, biofilm growth and its dispersion.

The interest in wastewater treatment processes using sedentary biomass (biofilms) was initiated by the Norwegian professor Hallvard Ødegaard, who was the first to use the moving bed biofilm reactor (MBBR). He assumed that biomass development would occur on elements which would move freely in the biological reactor. These elements were called the moving bed [10, 11].

The type of used filling has a significant impact on the efficiency of treatment using a moving bed. Most often, a selected natural or artificial material is used as the bed. Filling which consists of synthetic materials includes various types of loose fittings or packages (blocks) produced using polystyrene, polyvinyl chloride, polyamide, polypropylene, polyethylene, etc. Two basic types of packages are based on vertical and cross flow. In the deposit, the packages are placed at an appropriate angle in relation to the previous to ensure proper distribution of sewage. Synthetic deposits are characterized by a specific surface area up to 2000 m^2^/m^3^. Currently, carriers of various shapes and sizes are used. They should be designed to provide a high surface area to create optimal conditions for growth and maintaining proper biological membrane activity impossible [1–3].

The use of polymer composites with natural fillers, mainly wood-polymer composites (WPC) as carriers in the MBBR technology may prove to be an innovative solution, mainly due to the high active surface available for biological membrane development [12–16]. Due to the economic factor, i.e. increasing prices of raw materials for production of polymers (oil and gas), as well as the very high demand for polymer matrix composite materials, they have found application in an increasing number of industry sectors.

The dynamic development of the production of WPC results from the promising combination of properties of polymer components and natural additives. Ecological considerations play an additional role, because defective and post-use wood can be used to produce WPC [17–20]. The aim of the current research is to investigate the possibility of using molded WPC as a support in MBBR technology.

## Materials and method

**Fig. 1:**
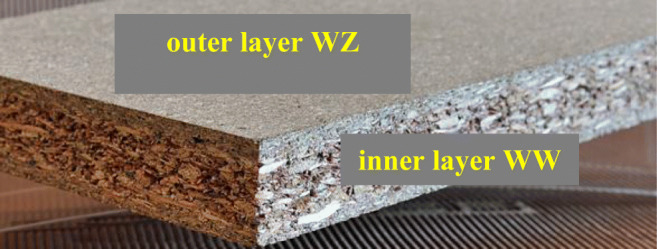
Places of sampling wood chips for testing inner layer - designation WW, outer layer - designation WZ

Two types of commercial wood flour, selected from conifers, were selected for the study: Lignocel C 120 with particle sizes in the range of 70 μm–150 μm and L9 with dimensions of 0.8–1.1 mm obtained from the German company J. Rettenmaier and Söhne GmbH CoKG, respectively. It was also decided to use wood chips used on an industrial scale for the production of chipboards. In the case of chipboard, the raw material was obtained as a result of cutting pine chips with ring cutters and, in order to crush large particles, it was subjected to the process of crushing and breaking in a hammer mill. Since the currently produced chipboard has a three-layer structure, the chips intended for individual layers are characterized by different dimensions. The smallest fractions (WZ) are used for the outer layers, and the largest (WW) for the inner layers (Fig. 1).

Therefore, in the carried out tests, WW and WZ type wood materials were treated as two separate groups of fillers, which were sorted using a vibrating shaker equipped with 5, 10, 18, 34, 60 mesh sieves. As a result, fractions with dimensions 0.25–0.5; 0.5–1.0; 1.0–2.0 mm were obtained for WZ chips, whereas for WW chips the fractions were characterized by dimensions in the range 0.5–1.0; 1.0–2.0 and 2.0–4.0 mm. The chips were characterized by high “slenderness”. Their aspect ratio was equal to approx. 15:1 for fine chips and 20:1 for large chips. Thus, 6 groups of wood chips were obtained, which were used to produce composites with a content 30% by weight of wood material. Polypropylene (PP) was used as the matrix for the Lignocel type composites with wood flour, while the selected WZ and WW chips were added to polyvinyl chloride (PVC). In the case of PP, Moplen PPH 648 T from Basell Orlen Polyolefines was used. It is an injection molding homopolymer which contains a nucleating and antistatic agent. It is characterized by very good fluidity and good stiffness.

In the case of PVC, it was a material obtained from a mixture produced by Anwil S.A. Włocawek, which contained poly(vinyl chloride) S-58, thermal stabilizers and lubricants with external and internal action. The starting materials for the production of composites were characterized in publications [13, 14, 18].

**Fig. 2:**
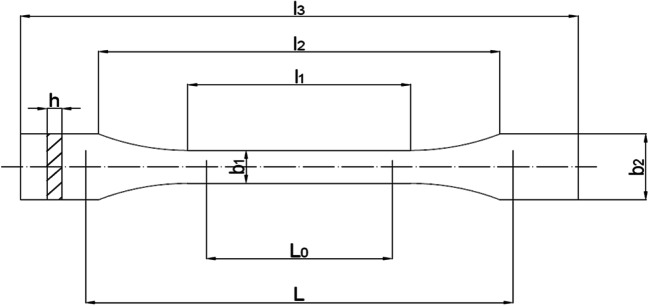
Test specimen. l_3_ – overall length, l_2_ – distance between wide, parallel parts, l_1_ – length of the part limited by parallel lines, b_2_ – width at the ends, b_1_ – width of the narrow part, h – recommended thickness, L_o_ – length of the measuring section, L – initial distance between the handles [Standard PN-EN ISO 527-2: 2012]

**Fig. 3:**
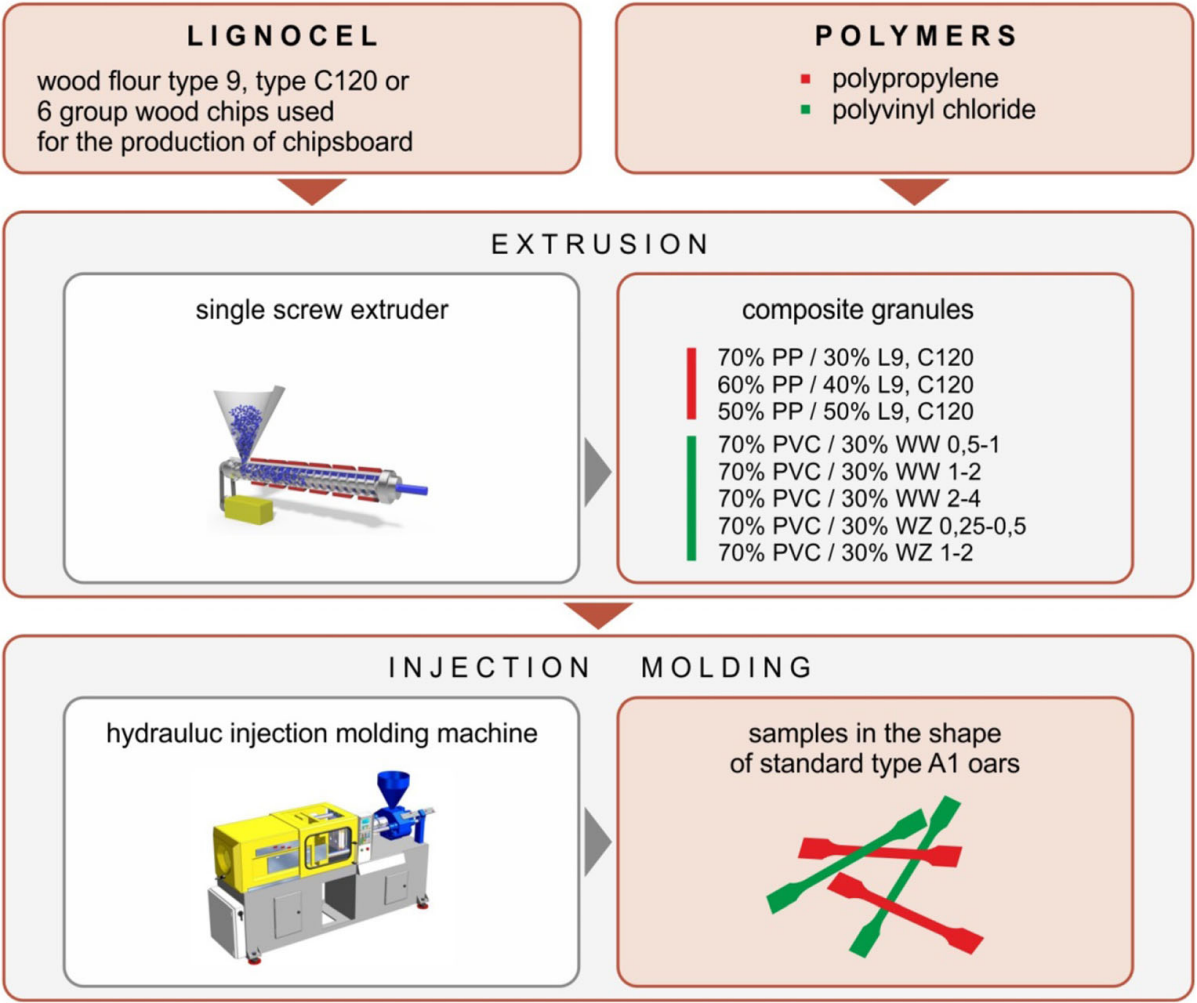
Stages of obtaining research samples

Homogenization of polymers with wood flour or wood shavings was carried out using a single-screw extrusion process, which resulted in the formation of composite granules. The test samples had the shape of standard type 1A oars (PN-EN ISO 527-2) (Fig. 2) and were manufactured by injection molding using a hydraulic injection molding machine model Wh–80Ap. The sample preparation scheme for testing is presented in Fig. 3. A typical sample (called a specimen in the standard) is flat and is characterized by a “paddle” shape. With a thickness of 4.0 ± 0.2 mm, the measuring portion width is equal to 10 ± 0.2 mm and the length is 80 or 60 mm.

In the next research stage, preliminary studies regarding the mechanical properties of the produced and selected composites were carried out, including the influence of the matrix and the type, quality, content of the filler and its type / species of wood, as well as the degree of its fragmentation, and the interaction at the matrix / filler interface [13, 16, 18] (Fig. 3).

The produced oars were placed in an aerated nitrification chamber of the bioreactor (6 m deep) located in the sewage treatment plant, with known technical and technological parameters. The composites were immersed in activated sludge up to a depth of 2.5 m and samples were collected every 3 months for microbiological tests. Microbiological tests were focused on the quantitative and qualitative assessment of microorganisms living on the composites. Three samples were collected from each system for testing. They were carried out using a Carl Zeiss Jena optical microscope with a 12.5x eyepiece, with 5x and 10x lens and a 1.5x magnification of the tube as well as using scanning electron microscopy (with a Jeo1JSM–7001F camera). The oars were placed in sterile bottles and then rinsed with 100 ml of sterile water. It’s known that activated sludge consists of aerobic and anaerobic microorganisms such as bacteria, archaea, fungi and protists. It is capable of degrading organic compounds which flow into the treatment plant. This method of collection allowed to obtain all types of microorganisms found on the surface of the composites, including settled, creeping and free-floating forms, as well as bacteria associated with the biofilm. The systematic affiliation of most microorganisms was determined based on microscopic observations [21, 22]. Determination of microorganisms was performed based on the generally available keys [23, 24]. In the case of microscopic analysis of bacteria, no diagnostic methods were used to determine their types or species, but attention was paid to the morphology of cells (filamentous and other, free-floating) [25, 26].

## Results

The number of microorganisms was determined by a modified estimation method using a 5–point scale: very numerous (5 points), numerous (4 points), moderately numerous (3 points), not very numerous (2 points) and single (1 point). The highest average number of detected organisms in the total research period was assumed to be 100%, while the remaining samples were estimated in relation to the maximum sample.

In the case of composites with a polyvinyl chloride matrix, it was found that ciliates (*Ciliata*), rotifers (*Rotifera*) as well as *Amoebozoa* were the most abundant on all composites, regardless of their type and amount of filler. Settled ciliates, represented by species belonging to genera *Epistylis* and *Opercularia coarctata* occurred permanently or periodically in high numbers. Free floating ciliates *Paramecium caudatum* were highly abundant, followed by amoebozoa represented by *Arcella* sp., rotifers (*Rotifera*) belonging to *Rotaria rotatoria* and other ciliates: *Vorticella microstoma*, *Carchesium polypinum*, *Litonotus* sp. and *Aspidisca* sp. The less abundant group included *Amoeba* sp., *Stentor* sp., *Tokophrya* sp. and flagellates (*Flagellata*) belonging to the *Peranema trichophorum* species. Occasionally, single representatives of the most common *Tardigradia*, *Gastrotricha, Aelosoma* sp. (*Oligochaeta*), nematodes (*Nematoda* n.det.) as well as *Cephalodella* sp., *Lecane* sp., *Glaucoma* sp. and *Trichodina pediculus* were found. Free-floating and filamentous bacteria were usually not very numerous or moderately numerous (Table 1). In contrast, in case of oars produced using pure, unfilled PVC, the amount of leached sludge (biofilm) was the lowest. Among the identified microorganisms, *Epistylis lacustris*, *Epistylis plicatilis, Epistylis chrysemydis, Opercularia coarctata, Paramecium caudatum*, and bacteria were not numerous. Other microorganisms, such as *Epistylis rotans*, *Epistylis coronata*, *Carchesium polypinum, Aspidisca* sp., *Peranema trichophorum*, *Rotaria rotatoria* as well as nematodes, gastropods and tardigrades occurred sporadically. The average density and average estimated number of determined microorganisms on all tested composites were summarized in Table 1.

**Table 1:**
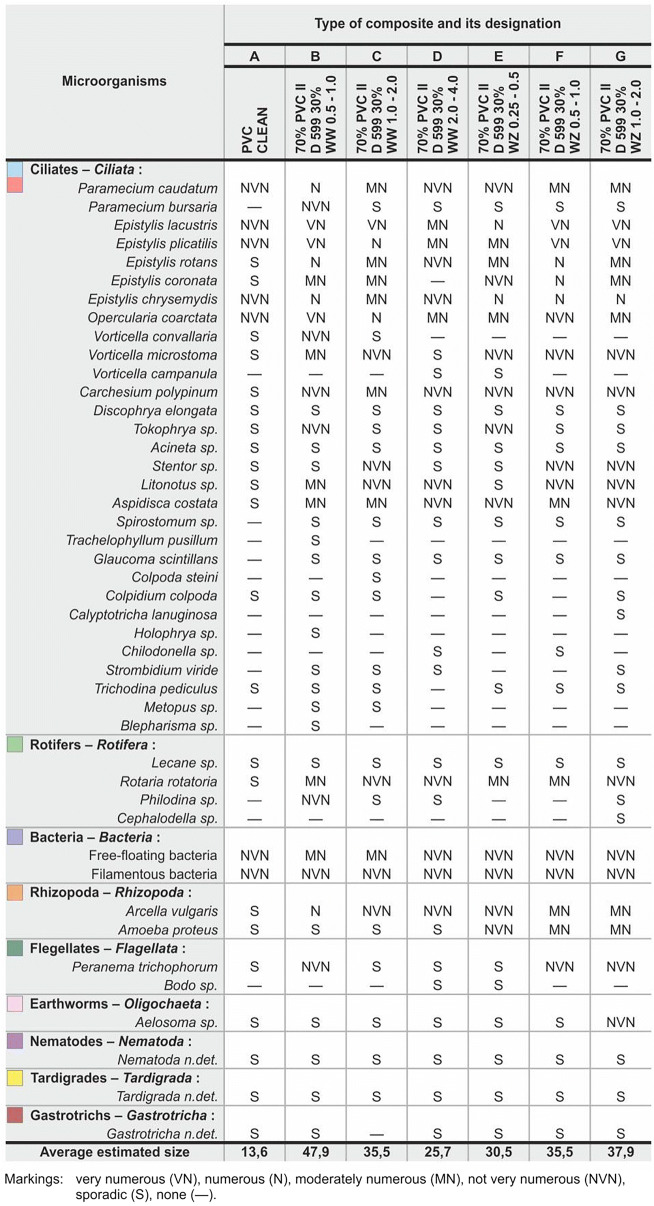
Average density and average estimated number of microorganisms which colonized the tested composites

All microorganisms which inhabited the composites were also found in the activated sludge collected directly from the nitrification chamber, in which the tested samples were suspended. Apart from studies regarding the identification of bacteria present in the activated sludge, which mainly participate in the processes of nitrogen compounds removal, research related to the determination of other microorganisms living in the activated sludge is still important. It mainly concerns ciliates, rotifers, amoebas and other microorganisms that are involved in the regulation of the number of bacteria and stimulate the formation of new bacterial generations.

During the microscopic analysis of activated sludge, fluctuations in the number of free-floating and filamentous bacteria were noted as well as sporadic occurrence of settled ciliates belonging to the *Stentor* sp. genera.

Based on the conducted research, it was established that the susceptibility of composites to biofilm formation and their colonization by microorganisms decreases with the increase of the size of WW wood particles. A different situation occurred in the case of composites containing WZ chips. In this case, the increase of chip size resulted in a significant increase of material susceptibility to biofilm formation was observed.

**Fig. 4:**
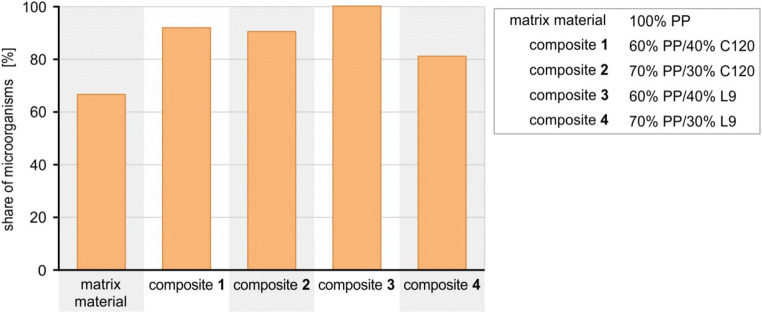
Estimated ratios of microorganisms deposited on the oars

The susceptibility of composites to the colonization by microorganisms is associated with different length to thickness ratio (aspect ratio) of WW and WZ type wood chips, which results from their different porosity after processing. As a consequence, the formed pores may be more or less susceptible to surface settlement by biological membranes, and these may form the basis for the settlement of organisms that are located further down the food chain. This hypothesis can be explained by the fact that a very similar average number of free-floating and filamentous bacteria associated with the layer of sediment present in the tested samples were determined for all composites. However, the amount of sediment on individual composites varied, and the remaining microorganisms in the sediment originally inhabited by bacteria occurred with a higher quantitative discrepancy. In addition, the higher ratio of chip length to thickness corresponds to lower surface, which results in lower susceptibility to microbial colonization of composites with WW chips. It should also be taken into account that during injection large wood particles are subjected to a much greater “compression” than small particles, due to pressure, which reduces the colonization area. In the case of polypropylene-based composites, analysis of the amount of biofilm-forming sludge over the entire study period (12 months) allowed to establish that the composite with 60% wt. of polypropylene PPH 648 T and 40% wt. of L9 type wood flour was the most susceptible to biofilm formation, followed by the composite which contained 70% wt. of polypropylene and 30% wt. of C120 type flour. The third material in terms of susceptibility consisted of 70% PPH 649 T and 30% wt. of L9, the fourth was 60% wt. of PPH 648 T and 40% wt. of C 120. Pure polypropylene PPH 648 T was the least susceptible material. The respective abundance of microorganisms was equal to: 2–91.8%, 3–90.3%, 4–81.0%, while the lowest density of organisms was observed in sample 5 (PPH 648 T) (66.6%) (Fig. 4).

The abundance of microorganisms occurring on the surface of all tested oars which consisted of a PP matrix and pure PP composites was strongly influenced by cocci, which mostly settled on the samples, as well as rotifers (*Rotaria rotatoria*) and amoebas (*Arcella vulgaris*), although in many in cases their number in the activated sludge chamber was lower. This indicates the beneficial effect of the tested solid support on the long-term deposition of microorganisms. The presence of all microorganisms which colonized the composites was also found during periodic testing of activated sludge occurring in the nitrification chamber in which the tested oars were suspended.

**Fig. 5:**
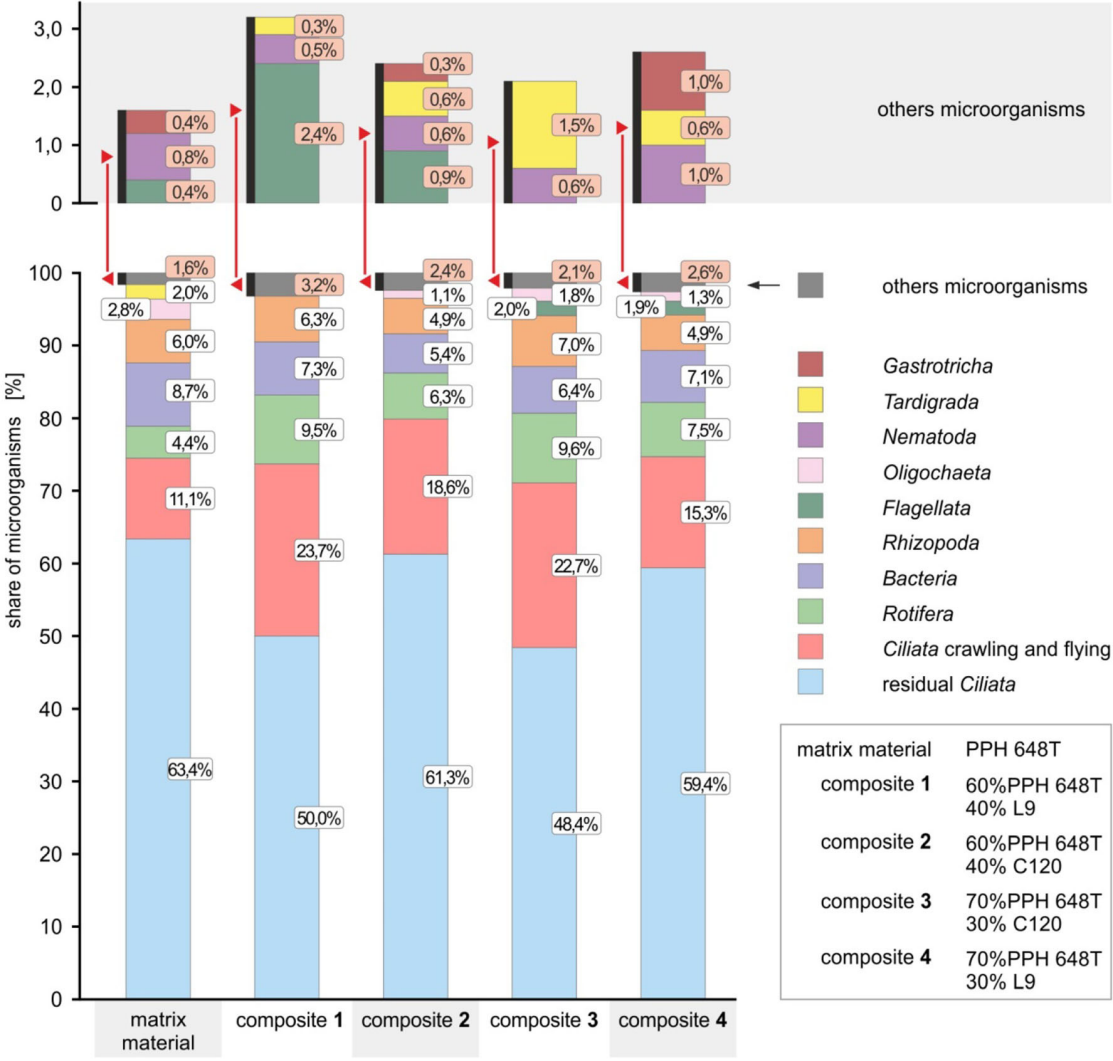
The ratios of microorganisms detected on the composite oars

The average percentage of microorganisms found on oars which consisted of various WPC with a PP matrix was summarized in Figs. 5. The settled ciliates included *Epistylis, Opercularia, Carchesium, Vorticella, Discophrya, Tokophrya, Acineta* and *Podophrya*, while creeping and floating ciliates were represented by the other detected genera. The bacteria included in the figures included both free-floating and filamentous forms.

**Fig. 6:**
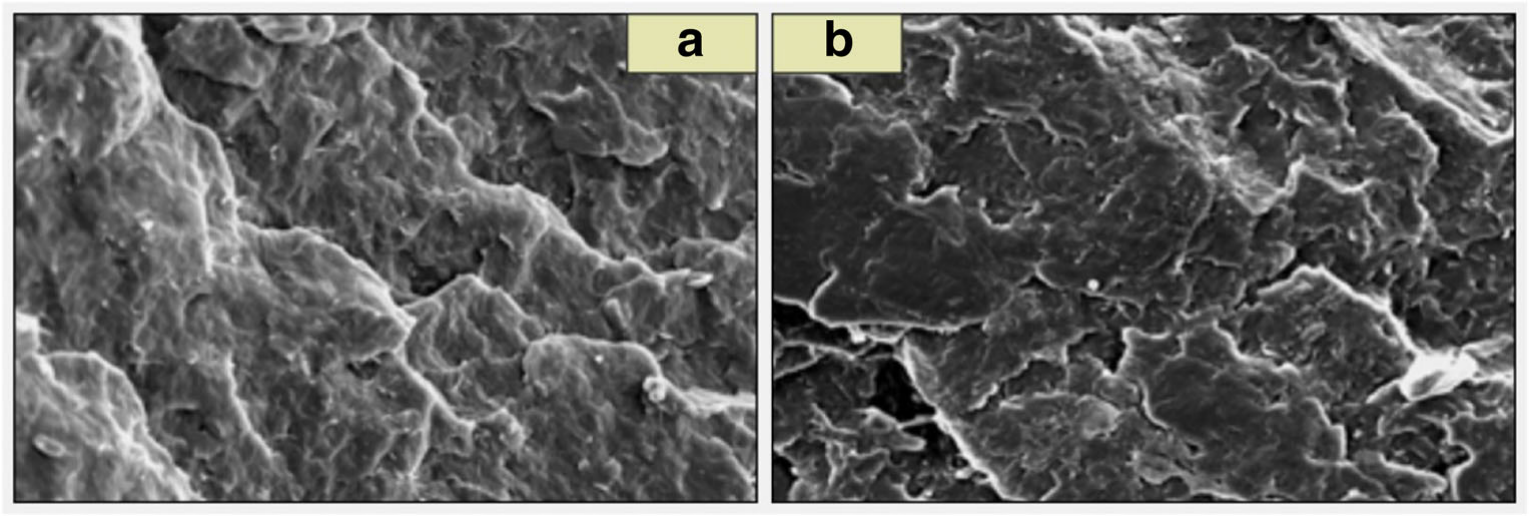
SEM images: **a** pure PPH 649 T before being placed in the bioreactor, **b** pure PPH 649 T after one year in the bioreactor

**Fig. 7:**
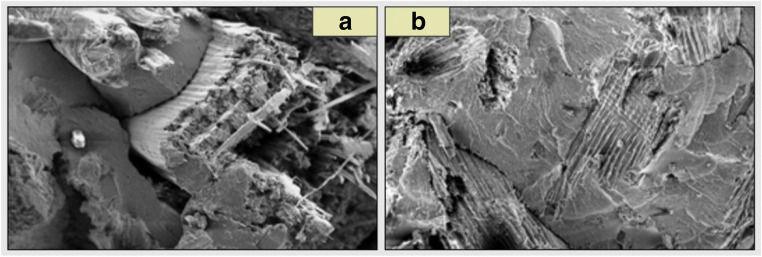
SEM images: **a** composite which consists of PPH 649 T and C120 flour before being placed in the bioreactor, **b** composite which consists of PPH 649 T and C120 flour after one year in the bioreactor

The studies regarding the composition of the formed biofilm using optical microscopy were supplemented with observations of the fractures of the tested composites carried out using scanning electron microscopy (with the Jeo1JSM-7001F apparatus). Images were obtained using the PC SEM Ver 2.1.09. software. In case of images obtained for pure polypropylene (Fig. 6a) and selected polymer wood composites (Fig. 7a), before placing them in the bioreactor, clear material structures can be observed. In the case of WPC, clear boundaries between wood chips and the polymer matrix are visible. In the polymer-wood composite a part of the filler is not covered with polymer, which promotes the development and growth of microorganisms (Fig. 7a).

In case of the images taken after the materials were kept in the bioreactor for 12 months (Figs. 6b and 7b), these borders are blurred. This is due to the interaction of microorganisms on the surface of the oars.

## Discussion

Based on the conducted research, it was established that the 12–month residence time of the oars in the activated sludge did not contribute to the destruction of the material.

During the microscopic analysis of microorganisms which resided on the surface of the tested samples, it was noticed that they most often occurred on 70% PVC II D 599 30% WW 0.5–1 and 70% PVC II D 599 30% WZ 1–2 composites. Ciliates and rotifers as well as amoeba occurred most commonly. The quantitative and qualitative ratios of microorganisms which inhabit the surfaces of composites were very similar to that of the activated sludge in the nitrification chamber. The conducted observations also indicated that the increase of chip sizes of a given WW type fraction resulted in decreased susceptibility of composites to biofilm formation. A different situation occurred in the case of composites containing WZ chips, since there was a significant decrease in material susceptibility to colonization by microorganisms along with the increase of filler size. In the case of polypropylene-based composites, analysis of the amount of biofilm-forming sludge over the entire study period (12 months) revealed that the composite with 60% wt. of PPH 648 T polypropylene and 40% wt. of L9 type wood flour was characterized by the highest average affinity for the deposition and growth of the biological membrane, followed by the composite which contained 70% wt. of polypropylene and 30% wt. of C120 type flour. The third material in terms of susceptibility consisted of 70% PPH 649 T and 30% wt. of L9, the fourth was 60% wt. of PPH 648 T and 40% wt. of C 120. Pure polypropylene PPH 648 T was the least susceptible material. The forces of biofilm adhesion to fittings affect the number of microorganisms and, consequently, the performance of the purification system. Based on the conducted research, it can be concluded that the use of properly selected polymer-wood composite fittings as a carrier will undoubtedly allow for the concentration of microorganisms in the bioreactor and thus, for the intensification of the purification processes. This can contribute to measurable environmental and economic effects in wastewater treatment processes. Ecological considerations also play an additional role, since defective and post-use wood can be used for WPC production.

## Conclusion

Various species of bacteria play a crucial role in both the degradation of organic compounds and the formation of the flocculent structure of the sediment. The physicochemical composition of wastewater that flows into the treatment plant plays a very important role in the formation of activated sludge flocs and the taxonomic diversity of activated sludge microorganisms. The microorganisms present in activated sludge tend to settle on various carriers present in the activated sludge. The conducted experiments have shown that the microorganisms which inhabit the moldings based on various composites prefer those containing wood chips or wood flour, while they are much less deposited on pure PVC and PP polymers. Polypropylene PPH 648 T and 40% wt. of L9 type wood flour was the most susceptible to biofilm formation. Pure polypropylene PPH 648 T was the least susceptible material.

## Data Availability

Not applicable.
